# Development of Polylactic Acid Films with Alkali- and Acetylation-Treated Flax and Hemp Fillers via Solution Casting Technique

**DOI:** 10.3390/polym16070996

**Published:** 2024-04-05

**Authors:** Anamol Pokharel, Kehinde James Falua, Amin Babaei-Ghazvini, Mostafa Nikkhah Dafchahi, Lope G. Tabil, Venkatesh Meda, Bishnu Acharya

**Affiliations:** Department of Chemical and Biological Engineering, University of Saskatchewan, 57 Campus Drive, Saskatoon, SK S7N 5A9, Canada; anamol.pokharel@usask.ca (A.P.); kehinde.falua@usask.ca (K.J.F.); amin.babaei@usask.ca (A.B.-G.); ync980@mail.usask.ca (M.N.D.); lope.tabil@usask.ca (L.G.T.); venkatesh.meda@usask.ca (V.M.)

**Keywords:** acetylation, composite, flax fiber, hemp fiber, poly (lactic) acid

## Abstract

This study aims to enhance value addition to agricultural byproducts to produce composites by the solution casting technique. It is well known that PLA is moisture-sensitive and deforms at high temperatures, which limits its use in some applications. When blending with plant-based fibers, the weak point is the poor filler–matrix interface. For this reason, surface modification was carried out on hemp and flax fibers via acetylation and alkaline treatments. The fibers were milled to obtain two particle sizes of <75 μm and 149–210 μm and were blended with poly (lactic) acid at different loadings (0, 2.5%, 5%, 10%, 20%, and 30%) to form a composite film The films were characterized for their spectroscopy, physical, and mechanical properties. All the film specimens showed C–O/O–H groups and the π–π interaction in untreated flax fillers showed lignin phenolic rings in the films. It was noticed that the maximum degradation temperature occurred at 362.5 °C. The highest WVPs for untreated, alkali-treated, and acetylation-treated composites were 20 × 10^−7^ g·m/m^2^ Pa·s (PLA/hemp30), 7.0 × 10^−7^ g·m/m^2^ Pa·s (PLA/hemp30), and 22 × 10^−7^ g·m/m^2^ Pa·s (PLA/hemp30), respectively. Increasing the filler content caused an increase in the color difference of the composite film compared with that of the neat PLA. Alkali-treated PLA/flax composites showed significant improvement in their tensile strength, elongation at break, and Young’s modulus at a 2.5 or 5% filler loading. An increase in the filler loadings caused a significant increase in the moisture absorbed, whereas the water contact angle decreased with an increasing filler concentration. Flax- and hemp-induced PLA-based composite films with 5 wt.% loadings showed a more stable compromise in all the examined properties and are expected to provide unique industrial applications with satisfactory performance.

## 1. Introduction

In the category of complex materials, bioplastics represent diverse biomaterials, with the bulk of their components developed from repeating monomer units of biomass such as cellulose, starch, vegetable oils, and vegetable fats. Currently, polycaprolactone (PCL), polybutylene succinate (PBS), polyhydroxyalkanoates (PHA), polylactic acid (PLA), and polyhydroxy butyrate (PHB) are commonly reported biodegradable bioplastics whose popularity has drawn significant attention in recent years [1]. According to Asim et al. [2], the characteristics of bioplastics, such as degradability, are a function of the degree of crystallinity, environmental factors, production process, and filler properties in blends and composites. This imparts unique features and peculiar advantages to each bioplastic and its applicability. As precursors for bioplastics, starch and cellulose are widely used, benefiting from their affordability and biodegradability [3,4]. Notwithstanding, their poor mechanical properties in their natural state have been reported as major drawbacks. Another source, namely poly (lactic) acid, has promising properties and stands out as a versatile and multipurpose choice for packaging applications due to its biodegradability, renewability, non-toxicity, excellent insulating properties, and wider applicability [5]. Despite the remarkable advantages of PLA, utilizing it as a bioplastic is highly induced with numerous drawbacks. For instance, PLA is moisture-sensitive and deforms at high temperatures, which restricts its applicability to specific processes. To sidestep its limitations, plant-based fibers (e.g., sisal, flax, hemp, coir, jute, etc.) are incorporated with PLA and tailored towards the development of bioplastics [6]. However, the application of natural fibers to biodegradable plastics requires a significant understanding of the inherent characteristics (e.g., fiber length, fiber orientation, type of treatment, etc.) of the fibers. Typically, fiber orientation is assigned the isotropic and mechanical properties of fibers, whereas fiber treatments (e.g., ultrasonic, chemical) can increase fiber–matrix adhesion and reduce moisture absorption.

Hemp (*Cannabis sativa subspecies* L.) has been produced for millennia and used in several food and non-food applications [7]. Essential oils and other vital biproducts could be recovered from hemp plants according to the literature [8,9]. Unfortunately, hemp stalks and roots are created as waste and typically end up in landfills. Thankfully, researchers have taken a keen interest in initiating ways of converting these green wastes into value-added products [10,11,12]. Available reports revealed that the tensile strength (sometimes > 1000 MPa), high aspect ratio (549), low density, and stiffness of extracted hemp fibers could position them as a promising reinforcing material for biocomposites. Nevertheless, hemp fibers have non-uniform and non-smooth surfaces, a restricted processing temperature (<230 °C), and low resistance to water absorption [13]. Some authors have studied PLA/hemp composites or hemp with other bioplastics [14], but insight from these studies still demands intensive research to investigate how developed biocomposites are affected by factors such as the source of the fiber, extraction procedure, type of treatment, and type of technology for efficient processing.

Flax (*Linum usitatissimum* L.) has piqued the interest of users owing to the possibilities of creating highly valued products from it. Flax fiber has been deployed for composite development as a result of its mechanical strength, which is hugely ascribed to the significant presence of cellulose (64.1–75%), hemi-cellulose (11–20.6%), and lignin (2–30%) [15,16]. Although studies have reported on flax-based biocomposites, scanty reports are available on flax and poly (lactic) acid as a sustainable route to non-biodegradable plastics. Dog-bone-shaped PLA/flax was developed using compression molding [17]. The authors observed decreased mechanical properties as a result of the environmental conditioning (75% and 95% RH) of the PLA/flax composite samples. Slicing parameters such as the layer height, interfilament distance, number of layers, microstructure, and tensile properties of 3D-printed PLA/flax have also been investigated [18]. By application, Kandola et al. [19] deduced the importance of PLA/flax as an excellent material as a flame retardant. Generally, improved mechanical properties have been reported in PLA/flax composites [20]. In a very similar study, Laziz et al. [21] investigated the surface treatment of PLA/flax fiber. The authors reported better flexural strength, higher energy, and good surface properties. Generally, the poor matrix of fibers (e.g., hemp and flax) is a major concern, suggesting the need for surface modification or treatment that mitigates against poor mechanical strength and other barriers such as hydrophilicity. A plethora of physical treatment methods, such as autoclave treatment [22], corona treatment [23], and plasma treatment [24] have been widely explored. Also, acid treatment, acetylation, dewaxing, grafting, and co-polymerization have been utilized as means of chemical treatment to improve the surface properties of natural fibers [25]. Despite this, alkali treatment has gained traction compared to other fiber treatment methods even though acetylation also offers promising features in the compatibility of natural fillers within a polymer matrix [5]. To date, this has attracted few studies, especially in PLA-based hemp and flax biomass. Additionally, the mechanical characteristics of bioplastics are reported to be influenced by several parameters, which are not limited to the type, weight, and size of the fillers used for their fabrication. Practical experiments are, however, scanty and those available either utilize other sources of natural fillers or biodegradable plastics different from PLA. To the best of our knowledge, this is the first study in which the treatment of the natural fillers (hemp and flax) and the effects of their weight (concentration) and sizes as processing conditions are investigated in the development of PLA-based films. We anticipate that this study should contribute to unraveling the mechanical characteristics of the films, thereby contributing to their usefulness in many applications.

The aim of this study is to develop PLA/alkali-treated/acetylation-treated hemp and flax composites and characterize them for their mechanical, thermal, color, and moisture properties. The findings from this work will further promote the importance of hemp and flax as a biomass for composite materials especially given the limited characterization studies on PLA/alkali-treated hemp and flax composites.

## 2. Materials and Methods

### 2.1. Materials

**Figure 1:**
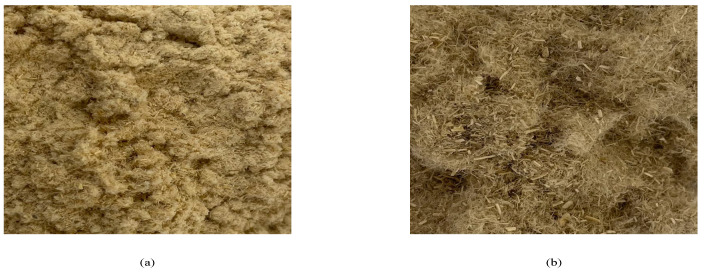
(**a**) Flax and (**b**) hemp (adapted from [5]). Scalebar: 1 cm.

Poly (lactic) acid (PLA 3D850^®^) filament was purchased from Natureworks, Plymouth, MN, USA. Flax and hemp fiber (*Katani cultivar*) were locally sourced from farmers in Saskatoon, SK, Canada, and KF hemp, respectively, as seen in Figure 1. Sodium hydroxide (NaOH), acetic anhydride, sulfuric acid, and chloroform were purchased from Sigma Aldrich^®^ Oakville, ON, Canada, and were used without any modification.

### 2.2. Method

#### 2.2.1. Alkali and Acetylation Treatment

For the alkali treatment, ~100 g pre-dried biomass was submerged in a 5% solution of sodium hydroxide at room temperature for 3 h. Thereafter, the biomass was thoroughly washed with distilled water until a pH of 7 was attained, thereby eliminating any residual alkali from its surface. The treated biomass was later dried at 105 °C in a thermogravimetric moisture test oven (Blue M^®^, Blue Island, IL, USA). For acetylation, ~100 g flax biomass was immersed in a glass beaker containing enough acetic anhydride to submerge it completely. To facilitate the reaction, about 5 mL sulfuric acid was added. After 15 min, the biomass was transferred to a reagent bottle and subjected to autoclaving at 121 °C to promote esterification. Finally, the treated biomass was thoroughly washed to eliminate residual chemicals until the pH reached 7, and then it was oven-dried at 105 °C.

#### 2.2.2. Milling Process

The pretreated biomass was milled using a knife mill (Retsch GmbH, SM1, Haan, Germany) automated with speed-controlling device. Thereafter, the resulting biomass particles were sieved using the Canadian Standard Sieve Series to obtain two different particle sizes: <75 μm and 149–210 μm. The procedure was such that the biomass was fed into the sieve with higher mesh opening and shaken to allow smaller particles to pass through the openings while the larger particles were retained on the sieve. This process was repeated until the desired particle sizes (<75 μm and 149–210 μm) were obtained.

#### 2.2.3. Preparation of PLA/Filler Films

The films were prepared using the solvent casting method. First, a liquor solution was prepared by dissolving PLA in chloroform at a 1:12 ratio using a laboratory magnetic stirrer. Then, biomass was added at varying concentrations (2.5%, 5%, 10%, 20%, and 30% weight based on PLA) to the liquor solution. The resulting mixture was thoroughly mixed for 48 h using a magnetic stirrer. The film-forming solution was then poured onto a glass plate (150 mm × 25 mm) and allowed to dry at room temperature. For comparison, neat PLA films without any fillers were also cast in a similar manner. The films were designated as “PLA/Filler name^Loading^”.

### 2.3. Characterization of the Film

#### 2.3.1. Fourier Transform Infrared Spectroscopy

The attenuated total reflectance-FTIR spectrophotometer (Spectrum 3 Tri-Range MIR/NIR/FIR Spectrometer, PerkinElmer, Shelton, CT, USA) was utilized to examine the FTIR spectra of the film samples. The analysis was carried out within the wavenumber ranges of 4000–650 cm^−1^, with a resolution of 4 cm^−1^.

#### 2.3.2. X-ray Photoelectron Spectroscopy

X-ray photoelectron spectroscopy (XPS) measurements were conducted utilizing a Kratos AXIS Supra system located at the Saskatchewan Structural Sciences Centre (SSSC). This system, produced by Kratos in Manchester, the UK, is equipped with a 500 mm Rowland circle monochromated Al K-α (1486.6 eV) X-ray source and features a combined hemispherical and spherical mirror analyzer (HSA/SMA). Analysis involved employing a hybrid slot with a 300–700-μ spot size. During the survey scan, binding energies were gathered within the range of 0 to 1200 eV in 1 eV increments, with a pass energy of 160 eV. High-resolution scans were carried out at intervals of 0.1 eV. The acquired data underwent analysis using CASA XPS software version 2.3.26 [26,27].

#### 2.3.3. Thermal Analysis

To evaluate the thermal stability of the film specimens, thermogravimetric analysis (TGA) was conducted using a thermogravimetric analyzer (Perkin-Elmer TGA 8000, Llantrisant, UK). Approximately 6 mg of the film sample underwent heating in the range of 50 to 900 °C at a heating rate of 20 °C/min, with a constant flow of nitrogen gas at 30 cm^3^/min. The differential thermogravimetric analysis (DTG) was derived by calculating the derivatives of the TGA data, facilitating the identification of the maximum temperature of disintegration at each phase of thermal degradation.

#### 2.3.4. Color

The color of the samples was measured with a portable colorimeter (WR10QC-8), which used the CIE standard illuminant D65 and a pointer from the CIE chromaticity diagram. Additionally, digital photographs were taken of the samples. The device then recorded the tristimulus color values of *L* (lightness), *a* (redness/greenness), and *b* (yellowness/blueness) at five random points on each film sample. The total color difference (ΔE), whiteness index (WI), and yellowness index (YI) were calculated using Equations (1)–(3), respectively.

(1) $$\Delta E={\left\{\left.{\left(\Delta L\right)}^{2}+{\left(\Delta a\right)}^{2}+{\left(\Delta b\right)}^{2}\right\}\right.}^{0.5}$$

(2) $$WI=100-{\left\{\left.{\left(100-L\right)}^{2}+{\left(a\right)}^{2}+{\left(b\right)}^{2}\right\}\right.}^{0.5}$$

(3) $$YI=\frac{\left(142.86\times b\right)}{L}$$

#### 2.3.5. Water Contact Angle

A sessile droplet method was used to determine the water contact angle of the films using a procedure described in [28]. Briefly, a tiny 5 μL droplet of distilled water was made on the surface of the film. Thereafter, a drop shape analyzer (AM2111, Dino-Light, Hsinchu City, Taiwan) was employed to capture the picture of the droplet. The contact angle, which is the angle formed between the baseline of the droplet and the tangent line at the point where it touches the surface, was then determined.

#### 2.3.6. Moisture Absorption

The moisture absorption was ascertained by comparing the weight of the specimens before and after conditioning at a humidity level between 50 and 55%. The test specimens (∅ 20 mm × 20 mm) were dried in a thermogravimetric moisture test oven (Blue M^®^, Blue Island, IL, USA) at 105 ± 1 °C until a constant weight was achieved. The specimens were carefully placed in a desiccator containing a saturated magnesium nitrate solution conditioned to ensure a humidity level of 53–55% at room temperature. Their weight was recorded at specific intervals until the specimen reached equilibrium. All the measurements were carried out in triplicates. The moisture absorption was evaluated using Equation (4):

(4) $$\mathrm{Moisture}\;\mathrm{absorption}=\frac{M2-M1}{M1}\times 100\%$$

where *M*1 and *M*2 are the initial and final mass (g) of the specimens, respectively.

#### 2.3.7. Water Vapor Permeability

To determine the water vapor permeability (WVP) of film specimens, the ASTM E96 standard [29] was used. The film specimens were conditioned for 48 h in a desiccator at 20 °C and 53–55% relative humidity. The film samples were affixed to the tops of glass vials containing dehydrated calcium chloride and the vials were tightly sealed with a screw cap that had a hole in the center. As a form of control experiment, three vials with an attached film specimen but without desiccant were used. The vials were kept in a desiccator with a more humid environment (75% RH). The weight of each vial and affixed film specimen was measured and recorded with an accuracy of 0.0001 g for 24 h. The weight of each vial and attached film specimen was recorded with precision, and the weight versus time plot was analyzed to calculate the WVP. The slope (S), which was estimated from the plot and the effective film area (A), was used to estimate the water vapor transmission rate (WVTR) and the water vapor permeability using Equations (5) and (6), respectively.

(5) $$WVTR=\frac{S}{A}$$

(6) $$WVP=\frac{WVTR\times X}{\Delta P}$$

where *X* and ΔP are the film thickness (m) and water vapor partial pressure (Pa), respectively.

#### 2.3.8. Mechanical Properties

The tensile strength (TS), elongation at break (EB), and Young’s modulus (YM) of the films were determined using an Instron Universal Testing Machine according to the ASTM D 882-88 standard [29]. A series of rectangular film strips (100 mm by 10 mm) were neatly cut. The machine was set in motion with an initial grip separation of 50 mm, moving with a steady crosshead speed of 75 mm/min utilizing a 1 kN load cell. The Young’s modulus (YM) was obtained from the slope of the initial portion of the stress–strain curve that was generated during the tensile test. To ensure accuracy, five replicates were performed for each film specimen, and *TS* and *EB* were calculated using Equations (7), (8), and (9), respectively.

(7) $$TS=\frac{F_{max}}{A_{min}}$$

(8) $$EB=\frac{L_{Max}}{L_{0}}$$

(9) $$YM=\frac{Stress}{Strain}$$

where *F_max_* represents the maximum force, *A_min_* denotes the minimum cross-sectional area, *L_max_* indicates the maximum elongation of the films after the load is applied to the samples, right up to the breaking point, and *L*_0_ is the initial length of the films before the load application. Stress is defined as the force applied per unit area of a material. Strain is defined as the change in length (deformation) of a material relative to its original length.

### 2.4. Statistical Analysis

After performing each test using an independently prepared film as the experimental unit, the results were then expressed as mean ± standard deviation (SD). To determine the significance of each mean value, a statistical analysis system was employed to perform one-way analysis of variance (ANOVA). Duncan’s multiple range test was then used to ascertain the statistical significance of each mean value, with a significance level of *p* < 0.05. Furthermore, to understand the effect of particle size, treatment, and loading of fillers and their interaction with the properties of the film, a three-way ANOVA was carried out.

## 3. Results and Discussion

### 3.1. FTIR

**Figure 2:**
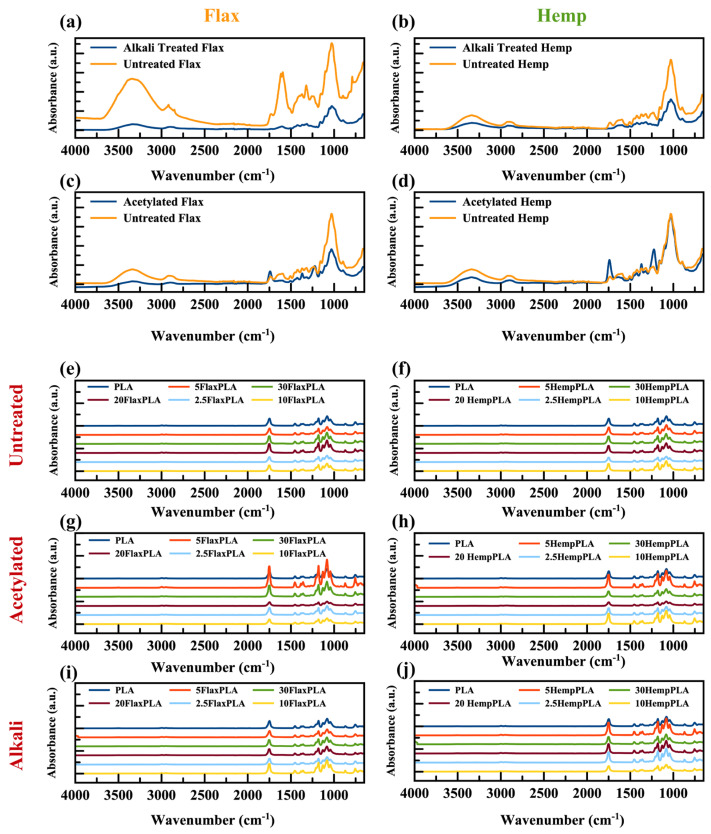
FTIR spectra of fillers and films. Flax fiber and hemp fiber (**a**–**d**). Control and composites containing treated and untreated fibers (**e**–**j**).

FTIR analysis was used to evaluate changes in the chemical composition of flax and hemp filler resulting from alkali and acetylation treatment [5]. As shown in Figure 2a–d, the peak at 1740 cm^−1^ in both the alkali- and acetylated-treated and untreated fillers is attributed to the C=O stretching of the acetyl or carboxylic acid groups of hemicellulose. However, the absence of this stretching after treatment is ascribed to the removal of hemicellulose. Nevertheless, the presence of the peak at approximately 1740 cm^−1^ in treated fillers indicates the potential formation of ester bonds between the acetyl groups and hydroxyl groups on the fillers. Additionally, the confirmation of ester bond formation is evident through the appearance of a novel peak at approximately 1229 cm^−1^, attributed to the C–O stretching of the ester carboxyl group. The broad peaks between 3320 cm^−1^ in the spectra are caused by the O–H groups of the fillers. The peak at 1232 cm^−1^, corresponding to the C–O stretching vibration in lignin, flattened after the treatment. These findings suggest that alkali treatment reduced the lignin content of hemp filler. A similar trend was observed in alkali-treated hemp fiber/natural rubber [23] and alkali-treated hemp fiber induced with polypropylene [30,31]. As observed by Fracz and colleagues, in most scenarios, lignin fuses the polysaccharide fibers together by filling the gaps between them [32]. Moreover, various absorption bands were identified in the pure PLA in Figure 2e–j. These included peaks at 3659 cm*^−^*^1^, indicative of the terminal O–H group, and at 2994 and 2946 cm*^−^*^1^, corresponding to the asymmetric and symmetric stretching vibrations of the CH_3_ groups in the side chains, respectively. The band at 1454 cm*^−^*^1^ was attributed to the bending vibration of C–H, while the pronounced absorption band at 1743 cm*^−^*^1^ denoted the stretching vibration of the carbonyl (–C=O) groups in the repeated ester units. Another intense absorption band at 1182 cm*^−^*^1^ was associated with the stretching vibration of C–O in CH–O within the polymer chains. Triplet peaks at 1130, 1082, and 1038 cm*^−^*^1^ represented C–O stretching vibrations in the C–O groups. Furthermore, absorption bands at 956 and 870 cm*^−^*^1^ were allocated to the C–C stretching of the single bond, and the robust absorption band at 749 cm*^−^*^1^ was ascribed to the deformation vibration of the CH_3_ groups. Generally, the PLA/fillers spectra were found to be like the PLA spectrum for all untreated, alkali-treated, and acetylation-treated samples, indicating that physical interactions were predominantly present without the formation of new functional groups. Similar FTIR results were reported in addition to fillers for PLA films in the literature [33,34].

### 3.2. X-ray Photoelectron Spectroscopy

**Figure 3:**
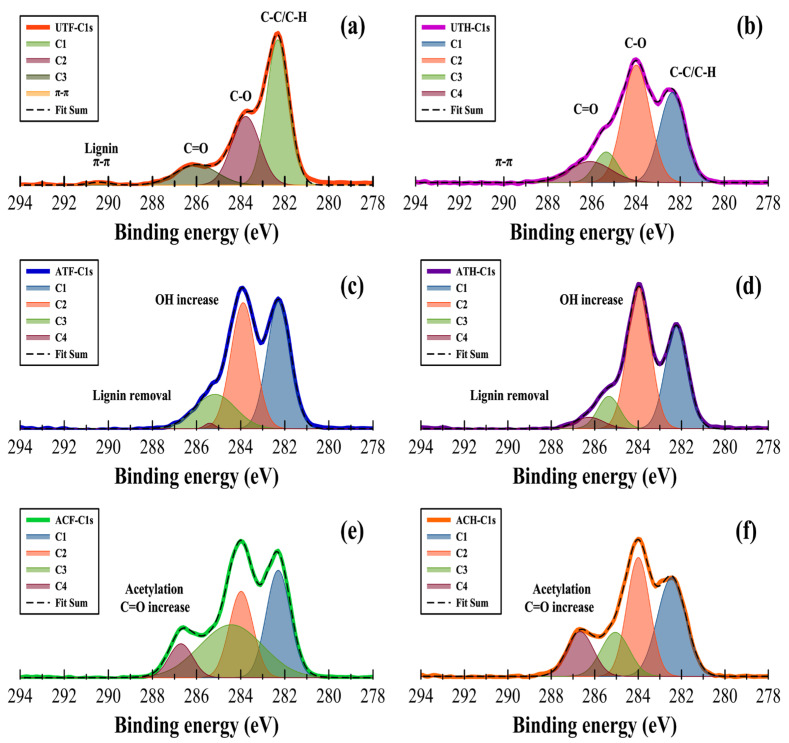
High-resolution XPS spectra. Signal assessments of different binding energies of (**a**) untreated flax, (**b**) untreated hemp, (**c**) alkali-treated flax, (**d**) alkali-treated hemp, (**e**) acetylated flax, and (**f**) acetylated hemp.

Untreated and treated flax and hemp fillers were investigated through X-ray photoelectron spectroscopy (XPS) to ascertain their elemental composition, atomic concentrations, and mass concentrations as seen in Figure 3a–f. C1 and C2 components were the primary constituents of the C1s spectrum for in the untreated, alkali-treated, and acetylated samples. The C1 peak of untreated flax and hemp had binding energies ranging between 284 eV and 282 eV. This confirms the presence of lignin and extracts (C–C/C–H). The C–O intensity of untreated hemp was significantly higher than that of flax. The π–π interaction in untreated flax fillers showed that lignin phenolic rings were present. Generally, C2, C3, and C4 were predominantly derived from cellulose [3,35,36] and hemicellulose. The decrease in the C1 intensity of alkali-treated flax indicated the effective removal of lignin in the flax by sodium hydroxide, whereas C2 showed a significant increase in their peaks owing to the emergence of hydroxyl groups on the surfaces (Figure 3c)**.** It is worth mentioning that the treatments (alkali and acetylation) eliminated the π–π interaction in the flax samples (Figure 3c,e). Higher O/C ratios indicated a significant carbohydrate content, while lower ratios suggested the presence of more lignin and extracts on the flax and hemp surfaces [37].

### 3.3. Thermogravimetric Analysis

**Figure 4:**
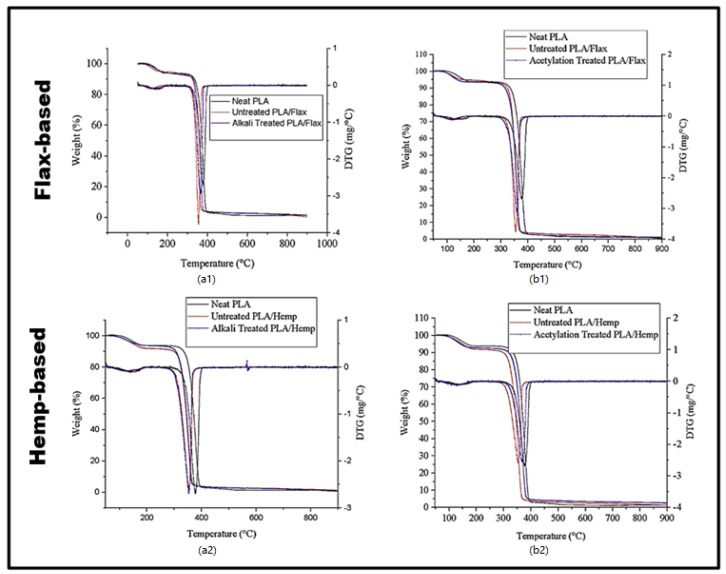
TGA and DTG of PLA/flax and PLA/hemp films with (**a1**,**a2**) alkali treatment and (**b1**,**b2**) acetylation treatment.

Thermogravimetric analysis was used to test the thermal stability of the neat PLA film and the PLA/filler composite film. The TGA and DTG thermograms showed that all the films displayed a two-step thermal degradation pattern, with the initial weight loss occurring near 100 °C (Figure 4). This is ascribed to solvent evaporation [5]. A drastic weight loss was observed in the second stage as the temperature increased from 270–410 °C. This is due to the rapid depolymerization of the polymers [38]. The thermal degradation of hemp and fiber-based composites has been previously reported and agreed with the degradation temperature reported in the present study [39,40]. Nonetheless, the maximum degradation temperature of the composite film’s occurred at 360 °C. Generally, both the neat PLA film and the PLA/filler composite film had the same maximum thermal decomposition temperature of 362.5 °C. This shows that incorporating the films with fillers did not significantly influence the overall thermal stability of the composite film in the present study. Several studies, as reported by some scholars, have also observed this trend in composite films [41,42,43]. These results suggest that PLA-based composites with fillers may be a practical option for various applications that necessitate both strength and thermal resistance, as the fillers do not seem to negatively impact thermal stability. A similar trend of thermal stability was observed in the PLA/curcumin film [33].

### 3.4. Color

**Figure 5:**
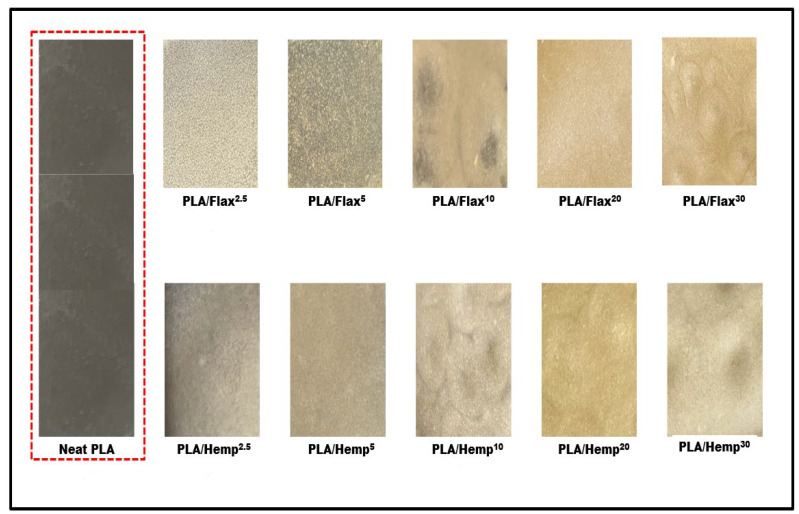
Color of PLA/flax and PLA/hemp films. Red dashed line was the neat PLA as the control sample.

By evaluating the color of the films, researchers can gain valuable insights into the effects of different treatments and fillers on the final product’s appearance. The results of the color test, as shown in Supplementary Tables S1–S4, show that the addition of both flax and hemp filler had a significant effect on the total color difference (ΔE), yellowness index (YI), and whiteness index (WI) values of the PLA film. The visual representations of the color changes observed in the PLA film with varying filler content are shown in Figure 5. Increasing the filler content caused an increase in the ΔE, resulting in a clear contrast from the original color of the PLA film. As the YI value increases, the yellowness of the film also increases. The observed results are in good agreement with previously reported results in PLA-based films using natural fillers [44,45,46]. The natural color of flax and hemp fillers could give a yellowish color to the final product. In contrast, the WI value decreases with the addition of flax and hemp filler, possibly due to the brown color of the fillers, which could darken the PLA film and reduce its overall whiteness. These findings suggest that the addition of fillers may not be desirable if the goal is to maintain the original color and whiteness of the PLA film. However, there may be certain applications where the use of flax filler could be beneficial, such as in packaging materials or in products where a natural, earthy look is desired.

### 3.5. Water Contact Angle

**Figure 6:**
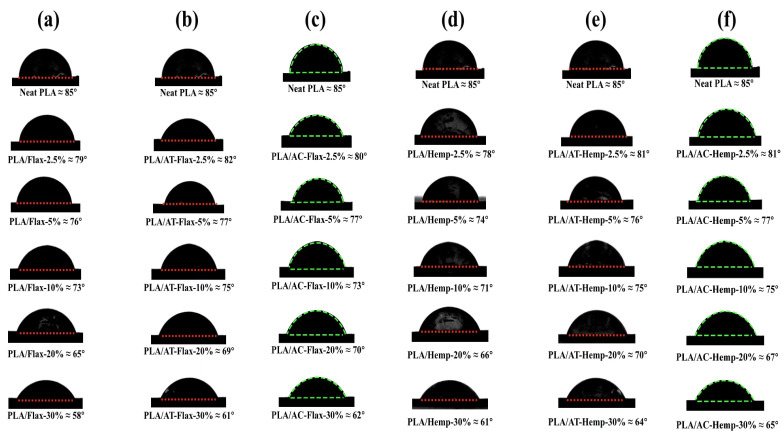
Water contact angle: (**a**) untreated flax/PLA, (**b**) alkali-treated flax/PLA, (**c**) acetylation-treated flax/PLA (green dashed line), (**d**) untreated hemp/PLA, (**e**) alkali-treated hemp/PLA, and (**f**) acetylation-treated hemp/PLA (green dashed line).

Fillers have an effect on the water contact angle (WCA) of PLA/flax- and PLA/hemp-based films. Generally, increasing the filler particle size caused no significant change in the WCA of the films regardless of their loadings and surface treatments. Compared with neat PLA with a WCA of 85°, blending PLA with a filler (hemp or flax) reduces the WCA. Figure 6 shows that the WCA of untreated, alkali-treated, and acetylation-treated PLA/flax varied between 79° and 58°, 82° and 61°, and 80° and 62°, respectively, as the filler content increases from 2.5 wt.% to 30 wt.%. Similarly, the WCA of untreated, alkali-treated, and acetylation-treated PLA/hemp varied between 78° and 61°, 81° and 64°, and 81° and 65°, respectively, as the filler content increased from 2.5 to 30 wt.%. In other words, the WCA decreases with increasing filler loadings. This suggests that increasing the filler particles tends to provide more sites for water molecules to interact with the polymer matrix, thereby reducing the contact angle. Additionally, the surface of the films became rougher in composites prepared with larger filler particles (149–210 μm) regardless of their treatments (Supplementary Materials, Tables S5 and S6).

### 3.6. Moisture Absorption

**Figure 7:**
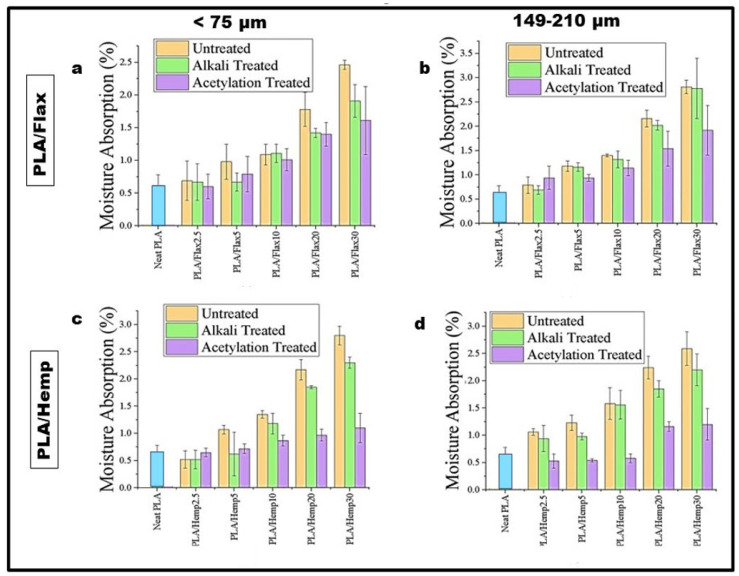
Moisture absorption of PLA/flax (**a**,**b**) and of PLA/hemp (**c**,**d**) films. The bar chart represents the difference between untreated, alkali-treated, and acetylation-treated fibers loaded into the PLA films.

Moisture absorption (MA) plays a crucial role in determining the performance and durability of bioplastics over an extended period. In the present study, the moisture absorption properties of PLA bioplastics with flax and hemp fillers were investigated for two filler particle sizes: <75 μm and 149–210 μm. For the PLA/flax composites, MA with filler content in untreated, acetylation-treated, and alkali-treated bioplastics increased significantly more than that of the neat PLA regardless of the particle sizes. Generally, the increase was more accentuated in the untreated bioplastics in most scenarios (Figure 7a,b). However, based on treatments, the moisture absorbed by the acetylation-treated PLA/flax composites were lower than that by the alkali-treated composites, except for PLA/flax5 at <75 μm and PLA/flax2.5 at 149–210 μm. As reported in past studies, alkali-treated fibers are generally low due to the removal of hemicellulose and lignin [47,48]. However, as a result of porosity, microcracks, and the complete relaxation of composites’ structures, the moisture absorption stages could be short and quick, slow and stable, and very quick [49]. By comparison, composites prepared with a 149–210 μm particle size tend to absorb more moisture than those prepared with a <75 μm particle size. It could be said that increasing the filler loadings resulted in an increase in the moisture absorbed by the composites due to the formation of hydrogen bonds within the fiber cell wall. Findings from [50,51] also validate this observation. Conversely, a higher filler particle size makes the material more porous and easier for moisture to penetrate the composites’ matrix [52,53,54]. Based on our investigation, we opined that the interplay between moisture absorption, loading percentage, and filler particles could be crucial in the development of PLA/flax composites. In the PLA/hemp composite prepared with a <75 μm filler particle size, the moisture absorbed in the untreated and alkali-treated composite showed a sharp reduction at 2.5% filler loadings, but at higher filler loadings, there was significant increase in the moisture absorbed (0.6–2.6%) (Figure 7c). Furthermore, at higher filler loadings (5–30%), there was a significant increase in the moisture absorbed. Using the 149–210 μm particle size, there was an initial decrease in the moisture absorbed by acetylated composites at 2.5%, 5%, and 10% filler loadings. This, however, was followed by a marked increase at 20% and 30% filler loads (Figure 7d). As reported in Asim et al. [2], fiber treatment enhances interfacial bonding and reduces moisture absorption. Notwithstanding, the influence of fiber concentration contributes to the moisture absorption in composites. At 40% fiber content, HDPE induced with 30% kenaf and 70% pineapple showed decreased overall water uptake [55]. The three-way ANOVA results indicate a statistically significant effect of particle size, treatment, and loading on the MA of both flax and hemp bioplastics. In the case of the flax bioplastic, the interaction between the particle size and loading was found to be significant for untreated filler, while it was insignificant for treated ones. For hemp bioplastic, the interaction between particle size and loading significantly affected the MA in untreated films, while this interaction was not significant in films with alkali-treated fillers. The results suggest that the method of treatment used on the fillers can have an influence on how the particle size and loading interact with each other, which ultimately affects the MA of the resulting films. These findings highlight the importance of considering multiple factors, such as particle size, treatment, and loading, when predicting the MA of polymer composites. The results of this statistical analysis can be used to develop effective strategies for mitigating moisture-induced degradation in bioplastics by optimizing the particle size, treatment, and loading of fillers.

### 3.7. Water Vapor Permeability

**Figure 8:**
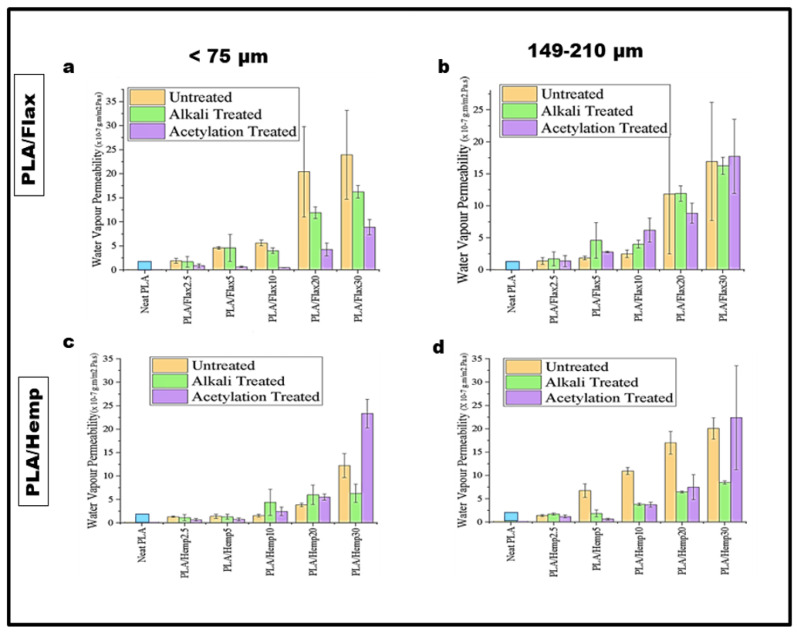
WVP of PLA/flax and PLA/hemp films. (**a**) PLA/Flax Particle size < 75 μm, (**b**) Particle size 149–210 μm, (**c**) PLA/Hemp Particle size < 75 μm, (**d**) Particle size 149-210 μm.

Water vapor permeability (WVP) is an important property to consider when designing bioplastics for packaging applications. The effects of filler loading, treatments, and particle size on the WVP of flax and hemp bioplastics were studied. For PLA/flax samples at a <75 μm particle size, the WVP of untreated and alkali-treated fillers increases with increasing filler loadings. Untreated PLA/flax30 and acetylation-treated PLA/flax10 had the highest (24 × 10^−^^7^ g·m/m^2^ Pa·s) and lowest (0.20 × 10^−^^7^ g·m/m^2^ Pa·s) WVP, respectively (Figure 8a). This observation, however, is in contrast with acetylation-treated composites at a filler loading of 2.5–10%, as the WVP of the samples reduced significantly. Buzarovska et al. [56] studied the WVP of neat PLA and PLA/talc composite films. The researchers found out that all PLA-based composite films had higher barrier properties (from 4.63 × 10^−^^12^ mol m/m^2^ sPa to 2.96 × 10^−^^12^ mol m/m^2^ sPa) than those of the neat PLA film (6.71 × 10^−^^12^ mol m/m^2^ sPa) and asserted that the crystallinity of PLA and the water vapor diffusion path could result in a decreased WVP. In the present study, higher filler loadings (20% and 30%) showed a marked increase in the WVP of acetylation-treated samples, with PLA/flax30 having the highest (9.5 × 10^−^^7^ g·m/m^2^ Pa·s) WVP. At a higher particle size (149–210 μm), the WVP of PLA/flax increases as the filler concentration increases (Figure 8b). Furthermore, higher filler loadings (20–30%) increase the WVP regardless of their treatments. Generally, a higher filler particle size (149–210 μm) at 2.5–10% filler loadings in an untreated composite resulted in lower WVP whereas higher WVP values were observed under these conditions in <75 μm particle size samples. In PLA/hemp composites, there was a reduction in the WVP of acetylated samples prepared with a <75 μm particle size at 2.5–5% filler loadings followed by a sudden increase at 10, 20, and 30% filler loadings (Figure 8c). As observed, the WVP of the composite is generally low (<5 × 10^−^^7^ g·m/m^2^ Pa·s) at 2.5–5% filler loadings regardless of the treatment. The reduction in WVP could be due to improved adhesion between the filler and matrix resulting from the treatments, reducing the number of voids between the filler and the matrix. Several other authors have reported improved barrier properties on PLA-based nanocomposites compared to those of neat PLA using a wide range of different treatments [57,58]. It was further confirmed in this study that at higher filler loadings (30%), the WVP of the composites increases in the order of acetylation-treated >> untreated > alkali-treated composites. The observed trend in WVP values for different particle sizes and filler loadings suggests that the clustering of filler particles is occurring at higher loadings, which can create voids or channels in the composite material that allow water vapor to pass through more easily. As shown in Figure 8d, the WVP of composites prepared with higher filler particle sizes (149–210 μm) was higher and increases at higher filler loadings regardless of the treatment. In most specimens, the WVP of the untreated composites was higher than that of its counterparts except in PLA/hemp30. The highest WVPs for untreated, alkali-treated, and acetylation-treated composites were 20 × 10^−^^7^ g·m/m^2^ Pa·s (PLA/hemp30), 7.0 × 10^−^^7^ g·m/m^2^ Pa·s (PLA/hemp30), and 22 × 10^−^^7^ g·m/m^2^ Pa·s (PLA/hemp30), respectively. The results of the statistical analysis showed that each factor, as well as the interaction between treatment and loading, had a significant effect on the WVP of the flax bioplastic films. This indicates that these factors individually or in combination affect the WVP values of the films. Additionally, the significant interaction between loading and treatment implies that the effect of loading on WVP is dependent on the treatment applied to the filler. The interaction between particle size and treatment and particle size and loading were not significant, indicating that the impact of particle size on WVP is independent of treatment or loading and vice versa. Similarly, the results of the analysis of the hemp bioplastic films indicated that each factor had a significant impact on the WVP of the films, and the interaction between these factors was also found to be significant. This suggests that the combined effect of particle size, loading, and treatment was greater than the effect of each factor individually.

### 3.8. Tensile Strength (TS), Elongation at Break (EB), and Young’s Modulus (YM)

**Figure 9:**
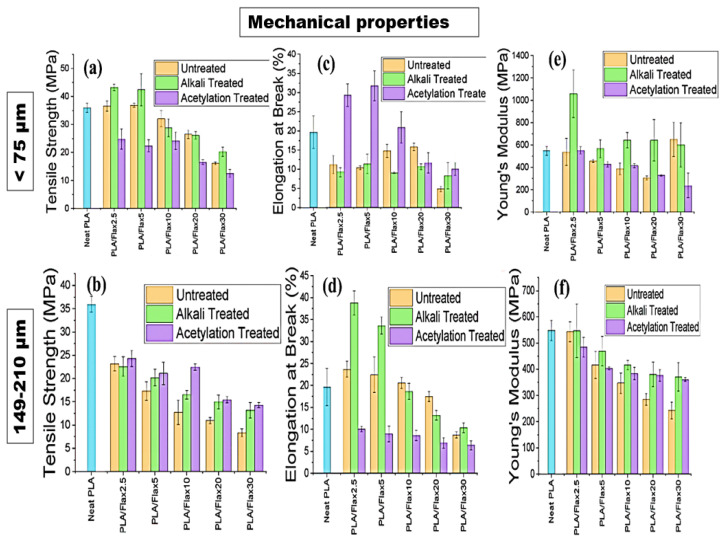

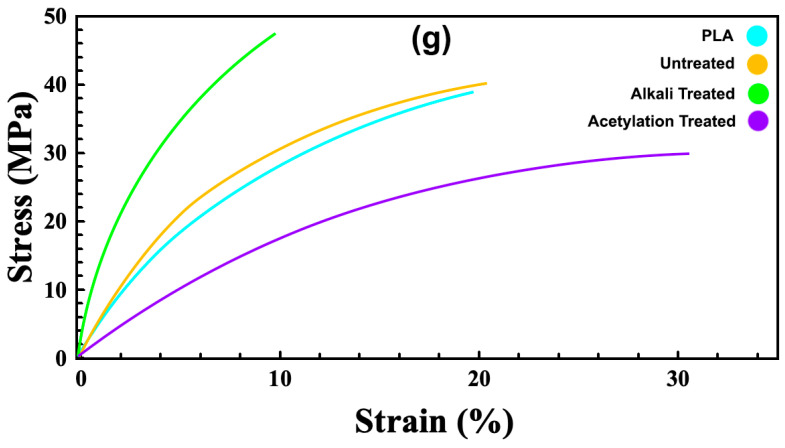
Mechanical characteristics of PLA/flax films. Particle size < 75 μm: (**a**) tensile strength, (**c**) elongation at break, and (**e**) Young’s modulus. Particle size 149–210 μm: (**b**) tensile strength, (**d**) elongation at break, and (**f**) Young’s modulus. Particle size < 75 μm: (**g**) stress–strain curves of PLA/flax films.

**Figure 10:**
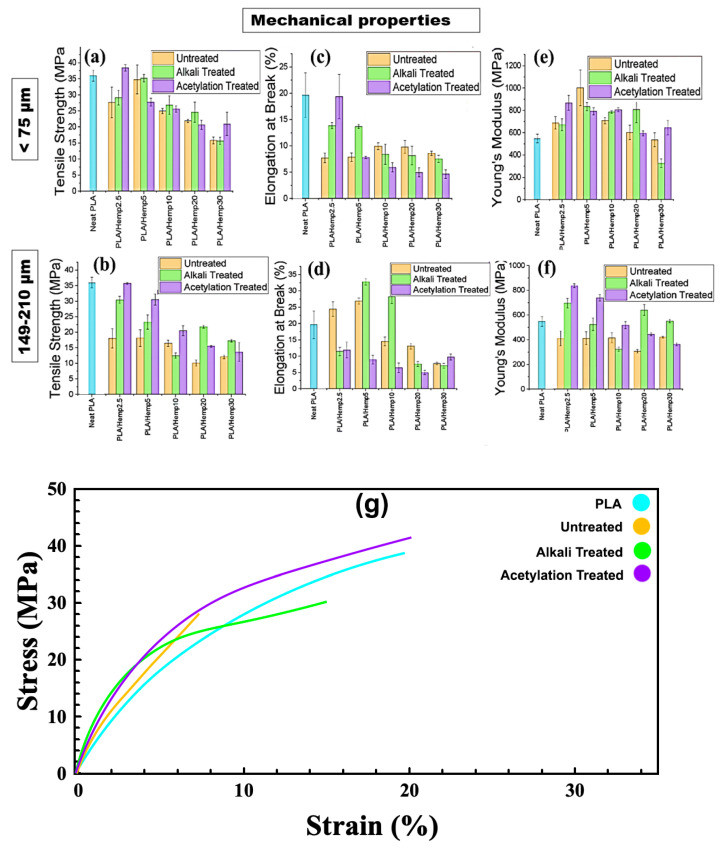
Mechanical characteristics of PLA/hemp films. Particle size < 75 μm: (**a**) tensile strength, (**c**) elongation at break, (**e**) Young’s modulus. Particle size < 149–210 μm: (**b**) tensile strength, (**d**) elongation at break, (**f**) Young’s modulus. Particle size < 75 μm: (**g**) stress–strain curves of PLA/hemp films.

The effects of filler particle size, filler loadings, and surface treatment were studied on the tensile strength (TS), young modulus (YM), and elongation at break (EB) of the flax-based and hemp-based bioplastics as shown in Figure 9a–f and Figure 10a–f, respectively. Generally, the particle size had no significant effect on the TS value (35 MPa) of the neat PLA regardless of their particle sizes and treatments. In Figure 9a, incorporating flax2.5 and flax5 into PLA increased the TS of untreated and alkali-treated specimens. A further increase in the particle loadings (10–30) resulted in a significant decrease in the TS value of the samples. Acetylation-treated composites showed a decrease in the TS between 22–25 MPa. In most cases, alkali-treated composites, except PLA/flax10, had a higher TS using the <75 μm particle filler. At a higher particle size (149–210 μm) (Figure 9b), acetylation-treated composites had a higher TS compared with that of the alkali-treated and untreated composites. Furthermore, an increase in the concentration of fillers resulted in a decrease in the TS for all the treatments except for acetylated PLA/flax10 where a higher TS value of 22 MPa was observed. Tested PLA-based composites stabilized with buckwheat husk showed a steady decrease in the value of strength at a filler content of 1% [59]. At more than 5wt.%, the TS of neat PLA and PLA/hemp reduced drastically due to the poor dispersion of fillers [60]. In most natural-fiber-reinforced composites, the decrease in TS and EB simultaneously corresponds to an increase in their modulus and a reduction in their strength is inevitable even if some modification is performed on them [61,62].

Obviously, besides modifying the mechanical properties of composites with fillers, other factors such as the concentration of fillers, dispersibility, production technique, and surface treatments are still crucial to the application of a high-performance composite. Figure 9c shows that acetylation-treated composites showed very low EBs (7–10%) compared with alkali-treated and untreated composites with EBs between 12 and 40% and 10 and 22%, respectively. Notwithstanding, increasing the weight of the fillers decreases the EBs regardless of the treatments. A decrease in elongation at break was also observed in reinforced epoxy composites using natural filler (walnut particles) [63] and PLA/cotton fabric biocomposites [64]. At a higher particle size of 149–210 μm, it could be said that acetylation improved the EBs of the composites in most scenarios except that of PLA/flax 20, where untreated composites had a higher EB (15%). Generally, variations in the concentration of the fillers resulted in unsteady trend of the EB values (Figure 9d). Using the <75 μm particle size, the results showed that the alkali-treated samples had a higher YM (>100 MPa) at a lower filler loading (PLA/flax2.5). However, a further increase in the filler loading drastically reduced the YM. The YM of untreated and acetylation-treated PLA/flax2.5 had a similar range to that of YMs with neat PLA. Further changes in the filler loadings, however, resulted in different YM values (Figure 9e). A more consistent trend was observed in composites prepared with 149–210 μm where alkali-treated samples outshined their counterparts in the YMs (Figure 9f). Other authors have demonstrated that filler size and types had a significant influence on the Young’s modulus of PLA/flax-based composites [65,66]. As observed by Batakliev et al. [67], the concentration of fillers had a significant influence on the young modulus of PLA-based nanocomposites. Notwithstanding, interfacial interactions through between the functional groups of fillers and composites may affect the Young’s modulus [68].

As seen in Figure 10a, alkali treatment improved the TS of PLA/hemp composites at lower filler loadings from 2.5% to 5% at a <75 μm particle size. However, an increase in filler particle sizes caused a significant reduction in the TS of all the composites regardless of their treatment and filler concentration. At a higher filler particle size, acetylation-treated composites had a higher TS than their counterparts at a filler concentration of 2.5–10 wt.% (Figure 10b). Acetylation treatment can improve the tensile strength of some composite materials [69,70]. Notwithstanding, excessive filler loading can lead to agglomeration, resulting in decreased mechanical properties [71,72]. The surface chemistry of composites influences their adhesion and interfacial bonding [73,74]. Similarly, past studies have also reported the effect of the surface area of the filler particles and their attributive effects on the strength of composites [75,76]. In the present study under consideration, the addition of filler particles reduces the mobility of the polymer chains, making the material more rigid and less able to stretch before failure, leading to a decrease in EBs at all filler loadings for a <75 μm particle size (Figure 10c). The natural fiber loading causes the PLA matrix’s structural integrity to be compromised, which accelerates fractures compared to a pure matrix and causes the composites’ decreased elongation at break [77,78]. Alkali treatment improves interfacial adhesion and increases elongation at break up to 5wt.% and 10 wt.%, but at higher loadings, agglomeration once again leads to defects and decreases the EBs as seen in Figure 10d. Past studies have reported improvements in the elongation at break of PLA/hemp because of alkaline treatment [78]. The YM of the composites increases with an increasing filler loading for <75 μm particle sizes in most samples. At a low filler loading of 2.5%, the acetylated-treated composite had a higher YM than that of the untreated and alkali-treated composites, but a further increase in the filler loadings decreases the YM (Figure 10e).

Furthermore, there is a significant increase in the YMs of untreated alkali at a filler loading of 2.5–5%, but a higher filler loading significantly decreases the YMs. For a 149–210 μm particle size, it could be inferred that treatment significantly affects the YM of the composite. At a lower filler loading (2.5%), acetylation-treated composites markedly showed a higher YM (500 MPa) with decreases with an increase in the filler loading from 5–20 wt.%. In contrast, while the alkali-treated composite initially showed a higher YM at a low filler loading of 2.5 wt.%, the YM reduces significantly between 5 and 10 wt.%. A further increase in the filler loading at 10 wt.% in the alkali-treated composite resulted in an increased YM, followed by a decrease at 20 wt.% (Figure 10f). Higher Young’s modulus values at low to moderate filler loadings are due to improved adhesion between the filler particles and the polymer matrix, resulting in better load transfer and reinforcement of the polymer. However, at higher filler loadings, the agglomeration of excess filler particles leads to a decrease in the Young’s modulus [68,79]. The three-way ANOVA results showed that all three factors, as well as their interactions, have a statistically significant impact on the TS, EB, and YM of the films. For a PLA/flax composite, the analysis revealed a statistically significant effect of particle size, treatment, and loading on TS, with the interaction between treatment and loading being significant for the filler of a particle size < 75 μm. However, the impact of the interaction between treatment and loading on TS was found to be insignificant for a particle size of 149–210 μm. For the EB and YM, a significant effect of particle size, treatment, loading, and their interaction was found. The size of the flax filler particles can have a significant impact on the YM of the composite material when untreated. However, when the particles are acetylated, their size does not seem to have a significant influence.

### 3.9. Material Chart

**Figure 11:**
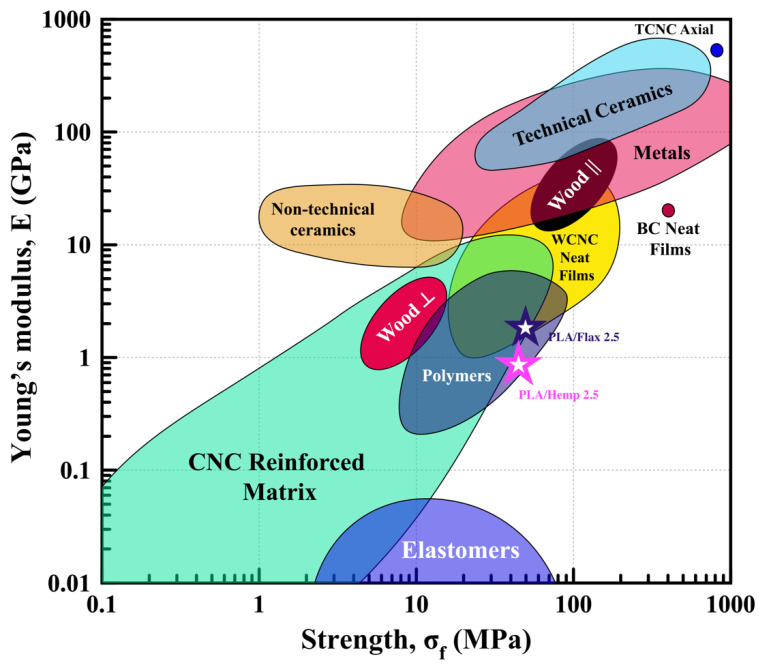
A chart displaying the properties of natural and synthetic materials is presented, with Young’s modulus plotted (adapted and modified from [4]). Values obtained in this study are denoted by stars.

Experimental measurements are used to compare the YM and TS of various materials, including flax- and hemp-based PLA composite films. This comparison is visualized through “Ashby plots,” where each region corresponds to a specific material class such as metals, ceramics, or wood [80]. As shown in Figure 11, properties of different material systems based on empirical data could be categorized and analyzed. Using this plot in the present study shows that PLA films incorporating 2.5% alkali-treated flax and hemp outperformed other blends in terms of mechanical properties. Notably, these formulations fall within the polymer region, highlighting their potential for developing composites with a high YM and TS.

## 4. Conclusions

Flax and hemp were modified and used as fillers for PLA-based composites at different filler loadings (0–30%) prepared by the solution casting technique. Spectroscopic analysis confirms the presence of C–O/O–H groups. Also, the π–π interaction in untreated flax fillers showed lignin phenolic rings in the polymeric behavior of the films. The influence of the fillers in the composites was not obvious during thermal decomposition since both specimens (neat PLA and composites films) had a degradation temperature of 362.5 °C. Increasing the filler content caused an increase in the color difference resulting in a clear contrast from the original color of the PLA film. Although the water contact angle decreases significantly as the filler loading increases, an increase in the moisture absorption and barrier properties against water uptake suggests good packaging potential for the films. Alkali-treated PLA/flax composites showed significant improvement in their tensile strength, elongation at break, and Young’s modulus at a 2.5 or 5% filler loading. Blending PLA with natural fillers such as hemp and flax can improve numerous properties of biocomposite film, but the interplay between several factors such as filler loadings and surface medication and type contributes significantly to these properties. Alkali- or acetylated-treated bioplastics employing natural fillers are environmentally viable alternatives to traditional plastics. However, future studies are required to determine the optimal processing conditions for their fabrication and to investigate their biodegradability and potential environmental impact.

## Data Availability

The raw data supporting the conclusions of this article will be made available by the authors on request.

## Supplementary Materials

The following supporting information can be downloaded at https://www.mdpi.com/article/10.3390/polym16070996/s1, Table S1: Color analysis of PLA/flax films (particle size < 75 μm); Table S2: Color analysis of PLA/flax films (particle size 149–210 μm), Table S3: Color analysis of PLA/hemp films (particle size < 75 μm), Table S4: Color analysis of PLA/hemp films (particle size 149–210 μm), Table S5: Water contact angle of PLA/flax films, Table S6: Water contact angle of PLA/hemp films, Table S7: Biochemical composition analysis (wt.%). References [81,82,83] are cited in the Supplementary Materials.
