# Supplementary material for: Development of Polylactic Acid Films with Alkali- and Acetylation-Treated Flax and Hemp Fillers via Solution Casting Technique
Source: Polymers (Basel). 2024 Apr 5;16(7):996. doi: 10.3390/polym16070996 (PMC11013793; doi:10.3390/polym16070996)
Supplement: Supplementary file 1 [file polymers-16-00996-s001.zip › polymers-2872476-supplementary.pdf]

SUPPLEMENTARY INFORMATION

Development of Polylactic Acid Bioplastic Films with Alkali Treated and Acetylation Treated Flax and Hemp Fillers via Solution Casting Technique

Anamol Pokharel, Kehinde James Falua, Amin Babaei-Ghazvini, Mostafa Nikkhah Dafchahi, Lope G. Tabil, Venkatesh Meda, Bishnu Acharya*

Department of Chemical and Biological Engineering, University of Saskatchewan, 57 Campus Drive, Saskatoon, SK S7N 5A9, Canada.

*Corresponding author: bishnu.acharya@usask.ca

Color of composite films

The results of the color test as shown in supplementary Tables S1, S2, S3, and S4 shows that the addition of both flax and hemp filler had a significant effect on the total color difference (ΔE), yellowness index (YI), and whiteness index (WI) values of the PLA film. Increasing the filler content caused an increase in the ΔE resulting in a clear contrast from the original color of the PLA film. As the YI value increases, the yellowness of the film also increases. The observed results are in good agreement with previously reported results in PLA-based films using natural fillers (Łopusiewicz et al., 2018; Olewnik-Kruszkowska et al., 2021, 2022; Roy & Rhim, 2021; Subbuvel & Kavan, 2022b, 2022a). The natural color of flax and hemp fillers could give a yellowish color to the final product. In contrast, the WI value decreases with the addition of flax and hemp filler, possibly due to the brown color of the fillers, which could darken the PLA film and reduce its overall whiteness. These findings suggest that the addition of fillers may not be desirable if the goal is to maintain the original color and whiteness of the PLA film. However, there may be certain applications where the use of flax filler could be beneficial, such as in packaging materials or in products where a natural, earthy look is desired.

Table S1: Color analysis of PLA/Flax films (particle size <75 μm)

Blends	Untreated			Alkali Treated		
	ΔE	WI	YI	ΔE	WI	YI
Neat PLA	9.85±1.42[e]	90.75±1.47[a]	−1.65±0.45[d]	9.85±1.42[f]	90.75±1.47[a]	−1.65±0.45[f]
PLA/Flax[2.5]	15.7±0.98[d]	83.81±1.06[b]	10.49±2.05[c]	13.45±0.47[e]	86.14±0.61[b]	7.62±2.28[e]
PLA/Flax[5]	19.71±4.28[d]	79.61±4.29[b]	17.34±4.07[c]	23.51±0.29[d]	75.48±0.3[c]	26.23±0.82[d]

PLA/Flax^{10}	31.49±1.55^{c}	67.65±1.52^{c}	34.99±1.42^{b}	31.26±0.94^{c}	67.61±0.87^{d}	40.83±1.54^{c}
PLA/Flax^{20}	44.16±3.04^{b}	55.18±2.3^{d}	48.92±7.11^{a}	43.29±4.3^{b}	55.82±4.29^{e}	55.52±8.86^{b}
PLA/Flax^{30}	51.15±2.96^{a}	48.38±2.93^{e}	48.79±6.05^{a}	49.93±0.97^{a}	49.28±0.96^{f}	65.45±1.73^{a}

‡ Means within each column with the same letters are not significantly different (P<0.05)

*Data are means ± SD

Table S2: Colour analysis of PLA/Flax films (particle size 149-210 μm)

Blends	Untreated			Alkali Treated		
	ΔE	WI	YI	ΔE	WI	YI
Neat PLA	9.85±1.42^{f}	90.75±1.47^{a}	−1.65±0.45^{f}	9.85±1.42^{f}	90.75±1.47^{a}	−1.65±0.45^{f}
PLA/Flax$^{2.5}$	18.36±0.51^{e}	81.2±0.5^{b}	10.81±0.28^{e}	16.75±0.81^{e}	82.93±0.82^{b}	7.34±1.37^{e}
PLA/Flax^{5}	25.32±1.73^{d}	73.98±1.7^{c}	21.94±1.21^{d}	24.1±1.96^{d}	75.24±1.92^{c}	18.83±0.79^{d}
PLA/Flax^{10}	33.31±3.4^{c}	66.04±3.44^{d}	30.42±6.14^{c}	30.81±1.4^{c}	68.3±1.33^{d}	32.97±0.4^{c}
PLA/Flax^{20}	38.94±0.49^{b}	60.43±0.46^{e}	37.88±2.97^{b}	40.93±1.24^{b}	58.19±1.24^{e}	49.88±2.56^{b}
PLA/Flax^{30}	47.8±0.32^{a}	51.62±0.3^{f}	49.12±0.57^{a}	47.94±1.55^{a}	51.23±1.51^{f}	62.53±1.59^{a}

‡ Means within each column with the same letters are not significantly different (P<0.05)

*Data are means ± SD

Table S3: Color analysis of PLA/Hemp films (particle size < 75 μm)

Blends	Untreated			Alkali Treated		
	ΔE	WI	YI	ΔE	WI	YI
Neat PLA	9.85±1.42^{f}	90.75±1.47^{a}	−1.65±0.45^{e}	9.85±1.42^{e}	90.75±1.47^{a}	−1.65±0.45^{e}
PLA/Hemp$^{2.5}$	22.68±1.39^{e}	76.74±1.46^{b}	16.64±2.78^{d}	19.28±0.64^{d}	80.21±0.62^{b}	12.27±0.33^{d}
PLA/Hemp^{5}	31.77±1.04^{d}	67.67±1.14^{c}	25.12±4.71^{c}	27.22±2.78^{c}	72.1±2.82^{c}	23.32±4.33^{c}
PLA/Hemp^{10}	39.4±0.4^{c}	59.97±0.39^{d}	37.7±0.7^{b}	34.9±0.41^{b}	64.34±0.38^{d}	35.32±2.94^{b}
PLA/Hemp^{20}	46.01±1.47^{b}	53.52±1.52^{e}	40.12±5.94^{b}	46.75±1.97^{a}	52.55±1.96^{e}	53.51±3.79^{a}
PLA/Hemp^{30}	60.32±0.55^{a}	39.33±0.57^{f}	57.77±5.33^{a}	45.66±0.92^{a}	53.83±1.05^{e}	41.37±8.41^{b}

‡ Means within each column with the same letters are not significantly different (P<0.05)

*Data are means ± SD

Table S4: Color analysis of PLA/Hemp films (particle size 149-210 μm)

Blends	Untreated			Alkali Treated		
	ΔE	WI	YI	ΔE	WI	YI
Neat PLA	9.85±1.42^{f}	90.75±1.47^{a}	−1.65±0.45^{f}	9.85±1.42^{f}	90.75±1.47^{a}	−1.65±0.45^{f}
PLA/Hemp$^{2.5}$	19.94±0.14^{e}	79.75±0.21^{b}	8.87±1.44^{e}	16.42±0.74^{e}	83.22±0.75^{b}	8.15±0.51^{e}
PLA/Hemp^{5}	25.33±1.94^{d}	74.11±1.92^{c}	18.35±0.98^{d}	24.37±1.22^{d}	75.02±1.28^{c}	18.33±2.84^{d}
PLA/Hemp^{10}	38.35±2.2^{c}	61.03±2.24^{d}	35.2±4.89^{c}	31.01±2.46^{c}	68.19±2.44^{d}	31.14±2.87^{c}
PLA/Hemp^{20}	43.17±1.64^{b}	56.25±1.68^{e}	41.51±4.48^{b}	36.83±2.43^{b}	62.43±2.49^{e}	38.2±7.37^{b}
PLA/Hemp^{30}	49.39±0.07^{a}	50.09±0.07^{f}	48.12±1.14^{a}	44.47±1.5^{a}	54.86±1.46^{f}	47.6±1.22^{a}

‡ Means within each column with the same letters are not significantly different (P<0.05)

*Data are means ± SD

Water contact angle

The WCA of untreated, alkali-treated, and acetylation-treated PLA/Flax varied between 79°-58°, 82°-61°, 80°-62°, respectively as the filler content increases from 2.5 wt.% to 30 wt.%. Similarly, the WCA of untreated, alkali-treated, and acetylation-treated PLA/Hemp varied between 78°-61°, 81°-64°, 81°-65°, respectively as the filler content increases from 2.5 wt.% to 30 wt.%. In other words, the WCA decreases with increasing filler loadings. This suggests that increasing the filler particles tends to provide more sites for water molecules to interact with the polymer matrix thereby reducing the contact angle

Table S5: Water contact angle of PLA/Flax films

Blends	75 μm		149-210 μm	
	Untreated	Alkali Treated	Untreated	Alkali Treated
Neat PLA	85.31±0.95^{a}	85.31±0.95^{a}	85.31±0.95^{a}	85.31±0.95^{a}
PLA/Flax$^{2.5}$	79.5±0.24^{b}	82±1.09^{b}	73.31±1.17^{b}	75.58±2.09^{b}
PLA/Flax^{5}	76.8±0.46^{c}	77.8±0.5^{c}	69.36±0.88^{c}	70.99±0.14^{c}
PLA/Flax^{10}	73.19±1.28^{d}	75.88±0.3^{d}	65.71±0.68^{d}	67.77±0.69^{c}
PLA/Flax^{20}	65.12±1.22^{e}	69.15±0.94^{e}	61.31±2.06^{e}	63.4±0.84^{d}
PLA/Flax^{30}	58.15±0.59^{f}	61.1±0.94^{f}	50.86±0.2^{f}	58.13±4.56^{e}

‡ Means within each column with the same letters are not significantly different (P<0.05)

*Data are means ± SD

Table S6: Water contact angle of PLA/Hemp films

Blends	75 µm		149-210 µm	
	Untreated	Alkali Treated	Untreated	Alkali Treated
Neat PLA	85.31±0.95[a]	85.31±0.95[a]	85.31±0.95[a]	85.31±0.95[a]
PLA/Hemp[2.5]	78.6±0.71[b]	81.15±1.12[b]	75.43±0.76[b]	77.22±0.72[b]
PLA/Hemp[5]	74.28±1.1[c]	76.97±0.96[c]	73.43±0.41[c]	75.55±0.98[c]
PLA/Hemp[10]	71.87±1.20[d]	75.2±0.62[c]	71.38±0.43[d]	72.62±0.61[d]
PLA/Hemp[20]	66.42±1.22[e]	70.85±0.81[d]	69.05±0.18[e]	70.01±0.79[e]
PLA/Hemp[30]	61.06±1.75[f]	64.98±2.07[e]	55.71±1.52[f]	62.23±0.83[f]

‡ Means within each column with the same letters are not significantly different ($P<0.05$)

*Data are means ± SD

Table S7: Biochemical Composition Analysis (wt.%)

Blends	Flax	Hemp
Cellulose	49.4	43.2
Hemicellulose	19.7	25.0
Lignin	20.5	23.0
Extractives and others	8.5	2.9

References

Łopusiewicz, Ł., Jędra, F., & Mizielińska, M. (2018). New Poly(lactic acid) Active Packaging Composite Films Incorporated with Fungal Melanin. In *Polymers* (Vol. 10, Issue 4). https://doi.org/10.3390/polym10040386

Olewnik-Kruszkowska, E., Gierszewska, M., Richert, A., Grabska-Zielińska, S., Rudawska, A., & Bouaziz, M. (2021). Antibacterial films based on polylactide with the addition of quercetin and poly (ethylene glycol). *Materials*, *14*(7), 1643.

Olewnik-Kruszkowska, E., Gierszewska, M., Wrona, M., Nerin, C., & Grabska-Zielińska, S. (2022). Polylactide-based films with the addition of poly (ethylene glycol) and extract of propolis—physico-chemical and storage properties. *Foods*, *11*(10), 1488.

Roy, S., & Rhim, J.-W. (2021). Antioxidant and antimicrobial poly (vinyl alcohol)-based films incorporated with grapefruit seed extract and curcumin. *Journal of Environmental Chemical Engineering*, *9*(1), 104694.

Subbuvel, M., & Kavan, P. (2022a). Development and investigation of antibacterial and antioxidant characteristics of poly lactic acid films blended with neem oil and curcumin. *Journal of Applied Polymer Science*, *139*(14), 51891.

Subbuvel, M., & Kavan, P. (2022b). Preparation and characterization of polylactic acid/fenugreek essential oil/curcumin composite films for food packaging applications. *International Journal of Biological Macromolecules*, *194*, 470–483.
